# Commentary: Physiological effects of bi-level high-flow nasal cannula in healthy individuals: a proof of concept trial

**DOI:** 10.3389/fmed.2026.1774347

**Published:** 2026-02-10

**Authors:** Adrián Gallardo, Armando Díaz-Cabrera, Mauro Castro-Sayat

**Affiliations:** 1 Servicio de Kinesiología y Cuidados Respiratorios, Sanatorio Clínica Modelo de Morón, Morón, Buenos Aires, Argentina; 2 Universidad Nacional de La Matanza, Departamento de Ciencias de la Salud, Kinesiología y Fisiatría, San Justo, Buenos Aires, Argentina; 3 San Juan de Dios Hospital, Santiago, Chile; 4 Faculty of Rehabilitation Sciences, School of Physical Therapy, Exercise and Rehabilitation Sciences Institute, Universidad Andrés Bello, Santiago, Chile; 5 Unidad de Soporte Ventilatorio No Invasivo (USoVNI), Hospital Juan A. Fernández, Buenos Aires, Argentina

**Keywords:** bi-level high-flow therapy, dead space washout, high-flow nasal cannula (HFNC), inspiratory effort, patient-ventilator interaction, respiratory mechanics

We read with great interest the study by Huh et al. (1), in which the authors present an intriguing technological innovation: the two-level high-flow nasal cannula (Bi-HFNC), designed to alternate between high inspiratory flow and low expiratory flow, potentially reducing expiratory resistance to improve comfort. While results in healthy individuals suggest improved subjective comfort and a reduction in nasopharyngeal PTP, there are critical physiological considerations, derived from the accumulated evidence on the use of HFNC, that we believe have not been fully addressed and that could limit its applicability in patients with acute respiratory failure (ARF). The applied technique is not equivalent to esophageal pressure measurement and could be considered as a potential methodological bias when extrapolating conclusions regarding true inspiratory muscle effort or work of breathing, particularly in pathological conditions.

We believe that the main limitation (at least theoretically) of Bi-HFNC lies in the reduction of flow during the expiratory phase. Current evidence supports the idea that one of the fundamental mechanisms of HFNC is the generation of positive end-expiratory pressure (CPAP-like effect) that optimizes end-expiratory lung volume (EELV) (2). Previous studies using electrical impedance tomography (EIT) have demonstrated that constant expiratory flow is directly responsible for increasing EELV and preventing alveolar collapse. Reducing expiratory flow introduce a potential drop in mean airway pressure, which could directly affect effective alveolar ventilation. In patients with poorly compliant lungs, even a momentary reduction in expiratory flow could result in insufficient alveolar recruitment and may impair the CPAP-like effect. The study by Huh et al. shows a reduction in expiratory pressure with Bi-HFNC; however, this “drop” could be counterproductive in clinical scenarios where stabilizing functional residual capacity is the primary therapeutic goal. If EELV decreases, oxygenation will be compromised, and the potential risk of patient-induced lung injury (P-SILI) could increase due to the increased inspiratory effort required to achieve optimal lung volume and increase oxygenation. Despite the above, it should be noted that P-SILI remains a largely theoretical construct, grounded primarily in indirect physiological and experimental evidence rather than in robust causal clinical data. The above is reminiscent of the effect sought with C-Flex, a system that modifies the CPAP effect during expiration by releasing continuous pressure during the expiratory phase. C-Flex demonstrated improved comfort in patients with chronic conditions such as obstructive sleep apnea, but not in cases of acute respiratory failure (3).

The second pillar of HFNC is dead space washout. This phenomenon depends on a flow that “sweeps” the exhaled gas away, replacing it with fresh gas. This process is fundamentally dependent on expiratory flow. Reducing the flow during exhalation in Bi-HFNC could decrease the efficiency of this washout. Evidence suggests that this washout is more effective when the flow is maintained high throughout the respiratory cycle (4). In this regard, a question not considered by the authors is whether reducing the expiratory flow allows for a residual accumulation of CO_2_ in the nasopharynx, which would increase rebreathing. This would also result in less effective alveolar ventilation, forcing the patient to increase their minute volume or respiratory rate, thus negating the benefit of reduced work of breathing (WOB) that has been reported with the therapy (5, 6). Nevertheless, in the absence of direct CO_2_ measurements, any inference regarding impaired washout under alternating flow conditions remains speculative and cannot be substantiated by the available data.

On the other hand, the use of a pressure trigger to alternate between the two flow levels is described. However, HFNC is, by definition, an open-flow system with constant leaks (allowing for open/closed mouth ambivalence). In patients with high ventilatory demand and tachypnea, the accuracy of a pressure trigger in an open system would therefore be highly questionable. Furthermore, the authors do not detail how the Bi-HFNC system manages variable oral leaks, which could generate erratic flow cycling—a manageable pattern under laboratory conditions in healthy volunteers, but difficult to control in an ARF environment with high ventilatory demand. In a critically ill patient with ARF, typically with increased ventilatory demand, an inspiratory-to-expiratory flow transition that is not perfectly synchronized with the patient's neural effort could generate an additional resistive load, worsening diaphragmatic fatigue instead of alleviating it. In this context, neural triggering strategies, such as synchronization via diaphragmatic electrical activity (EAdi) using a NAVA catheter, may represent a potential future solution to achieve appropriate cycling. While Huh et al. (1) report a decrease in inspiratory pressure balance is reported, this data should be interpreted with caution. In healthy subjects, lung mechanics are linear and predictable; however, during respiratory failure, work of breathing (WOB) depends not only on the inspiratory pressure required to overcome resistance but also on the elastic load imposed by the loss of lung volume. If Bi-HFNC reduces end-expiratory lung volume loss (EELV) (due to low expiratory flow), lung compliance could worsen significantly. Consequently, although the device facilitates high-flow air entry, the patient may require greater muscular effort to expand a lung that tends to collapse at the end of each expiration. Therefore, the net impact on total WOB could be negligible or even detrimental in critically ill patients.

For Bi-HFNC to move beyond being a “concept” and become a clinical tool, it is imperative that future studies address the following questions: How does alternating flow affect the regional distribution of ventilation and EELV compared to conventional HFNC? Is bi-HFNC capable of effectively maintaining O_2_ and CO_2_ levels in patients with moderate to severe hypoxemia? Optimizing oxygenation with HFNC appears to reach a therapeutic limit when the delivered flow rate is around 1.5 times the patient's peak inspiratory flow (7). In this situation, the flow rate exceeds inspiratory demand, so stabilizing FiO_2_ and CO_2_ washout hardly explains the occurrence of this plateau. In this context, it is plausible that the observed benefits are mainly linked to increased airway pressure. Since these mechanisms depend on the delivered flow rate, reducing the flow rate during the expiratory phase could compromise the achieved physiological effects without offering any additional sustainable benefits. Furthermore, what would be the incidence of cycling errors and ineffective triggers in the presence of significant oral leaks?

The study by Huh et al. (1) opens a necessary door toward personalized high-flow therapy and patient comfort. However, respiratory physiology suggests that the “price” of this hypothetical comfort could be a compromise in alveolar stability and dead space washout efficiency. In critically ill patients, oxygenation and effort reduction depend on a stable mean airway pressure. Consequently, Bi-HFNC must be rigorously validated before being considered a safe alternative to conventional HFNC.
